# Dispersal ability predicts spatial genetic structure in native mammals persisting across an urbanization gradient

**DOI:** 10.1111/eva.13133

**Published:** 2020-11-06

**Authors:** Jonathan L. Richardson, Sozos Michaelides, Matthew Combs, Mihajla Djan, Lianne Bisch, Kerry Barrett, Georgianna Silveira, Justin Butler, Than Thar Aye, Jason Munshi‐South, Michael DiMatteo, Charles Brown, Thomas J. McGreevy

**Affiliations:** ^1^ Department of Biology University of Richmond Richmond VA USA; ^2^ Department of Natural Resources Science University of Rhode Island Kingston RI USA; ^3^ Ecology, Evolution and Environmental Biology Department Columbia University New York NY USA; ^4^ Department of Biology and Ecology Faculty of Sciences University of Novi Sad Novi Sad Serbia; ^5^ Department of Biology Providence College Providence RI USA; ^6^ Health and Human Services Department City of Somerville Somerville MA USA; ^7^ Department of Biological Sciences Fordham University Bronx NY USA; ^8^ State Health Laboratory Rhode Island Department of Health Providence RI USA; ^9^ Division of Fish & Wildlife Rhode Island Department of Environmental Management West Kingston RI USA

**Keywords:** gene flow, genetic structure, landscape genetics, panmixia, restriction site‐associated DNA, small mammals, synanthropic, urban evolution

## Abstract

As the rate of urbanization continues to increase globally, a growing body of research is emerging that investigates how urbanization shapes the movement—and consequent gene flow—of species in cities. Of particular interest are native species that persist in cities, either as small relict populations or as larger populations of synanthropic species that thrive alongside humans in new urban environments. In this study, we used genomic sequence data (SNPs) and spatially explicit individual‐based analyses to directly compare the genetic structure and patterns of gene flow in two small mammals with different dispersal abilities that occupy the same urbanized landscape to evaluate how mobility impacts genetic connectivity. We collected 215 white‐footed mice (*Peromyscus leucopus*) and 380 big brown bats (*Eptesicus fuscus*) across an urban‐to‐rural gradient within the Providence, Rhode Island (U.S.A.) metropolitan area (population =1,600,000 people). We found that mice and bats exhibit clear differences in their spatial genetic structure that are consistent with their dispersal abilities, with urbanization having a stronger effect on *Peromyscus* mice. There were sharp breaks in the genetic structure of mice within the Providence urban core, as well as reduced rates of migration and an increase in inbreeding with more urbanization. In contrast, bats showed very weak genetic structuring across the entire study area, suggesting a near‐panmictic gene pool likely due to the ability to disperse by flight. Genetic diversity remained stable for both species across the study region. Mice also exhibited a stronger reduction in gene flow between island and mainland populations than bats. This study represents one of the first to directly compare multiple species within the same urban‐to‐rural landscape gradient, an important gap to fill for urban ecology and evolution. Moreover, here we document the impacts of dispersal capacity on connectivity for native species that have persisted as the urban landscape matrix expands.

## 1 INTRODUCTION

Total urban land cover across the world is projected to increase 185% between the years 2000 and 2030 (Seto et al., 2012), outpacing even the rate of human population growth (Liu et al., 2020). Despite this rapid urbanization, some native species have been able to persist at stable or even increasing numbers. However, there is little mechanistic understanding about the traits (e.g., behavioral, life history, body size) that promote persistence or decline of native species in and around cities (Lowe et al., 2017; Santini et al., 2019). It has been suggested that movement and dispersal ability are associated with persistence in urbanizing landscapes, as dispersing individuals can colonize new urban habitat or aid with demographic and genetic rescue in residual patches. Research has focused on the urban fragmentation of native habitats through the lens of metapopulation connectivity (Melero et al., 2020; Rochat et al., 2017; Wood & Pullin, 2002). Habitat fragmentation of residual natural habitats is clearly an issue for the persistence of many species (Tucker et al., 2018), but urbanization can also create new suitable habitat for some native species, perhaps fusing previously fragmented habitat (Menke et al., 2011) and facilitating movement of some species (Miles et al., 2018). In contrast to potential fragmentation or facilitation of dispersal, the movement of some species appears unaffected by cities, representing a third, null model (e.g., Theodorou et al., 2018). In the case of each of these models of urban impacts on connectivity (i.e., fragmentation, facilitation, or neutral), dispersal abilities may be tightly linked to persistence in or colonization of urban environments (Bierwagen, 2007; Rochat et al., 2017).

Native species that can take advantage of resources found in both remaining greenspaces and urban infrastructure likely have a significant advantage over species reliant on residual native habitats. Species with this flexibility have been characterized as "urban adapters" for their ability to facultatively use human‐subsidized food resources and buildings/structures for shelter (McKinney, 2002). The terms "synanthropic" and "peri‐domestic" have also been used in various ways for species that can live in close proximity to people without being obligately dependent on human resources (Hulme‐Beaman et al., 2016). Given their ecological flexibility, native synanthropic species are often the ones persisting in urbanized environments (Gliwicz et al., 1994; Gross et al., 2012; Withey & Marzluff, 2009). Furthermore, highly mobile synanthropic species will likely maintain connections with exurban populations. This connectivity has the potential to not only bolster the urban population through demographic rescue and an infusion of genetic variation (Gomulkiewicz & Holt, 1995; Gonzalez et al., 2013; Hufbauer et al., 2015), but also potentially slow the local adaptation to urban environments due to allele swamping and outbreeding depression (Haldane, 1930; Lenormand, 2002; Wright, 1931). Studies of the eco‐evolutionary outcomes of connectivity are critical for the burgeoning field of urban evolutionary biology, and characterizing dispersal and gene flow for species occupying the entire urban‐to‐rural gradient is a first critical step (Munshi‐South & Richardson, 2020).

In this study, we focus on two synanthropic species that inhabit the same urban‐to‐rural gradient in cities throughout North America, yet have very different dispersal capacities—white‐footed mice (*Peromyscus leucopus*) and big brown bats (*Eptesicus fuscus*). Both species are found within large forested, field and edge habitats, which are their native habitats. But they are also common within the entire range of human‐developed landscapes, including the urban core. While bats can roost in nearly any vertical structure within a city (personal observations), urban white‐footed mice are more likely to be found in patches of secondary forest with canopy cover, as well as areas with thick vegetative ground cover (Barko et al., 2003), such as urban parks and greenspaces (Munshi‐South, 2012). Despite both species occupying the full urban‐to‐rural gradient, white‐footed mice rarely disperse more than 500 meters (Stickel, 1968), with home ranges limited to around 1,000 m^2^ (Teferi & Millar, 1994), and much less in urban areas. Meanwhile, bats consistently exhibit much larger home and dispersal ranges, from a daily foraging range of 1.8 km in big brown bats (Brigham, 1991) to a maximum dispersal event of 74 km in some species of fruit bat (Abedi‐Lartey et al., 2016; Tsoar et al., 2010). We use these disparities in dispersal ability to generate hypotheses about the expected degree of connectivity for these mice and bats across a focal landscape (see below).

While movement and dispersal in and around cities are of primary interest to urban ecologists, they are difficult to measure directly for individual organisms, making genetic‐based proxies of connectivity necessary. There is long‐standing theory indicating that dispersal ability (i.e., vagility) should be tightly associated with levels of gene flow and genetic structuring over space (Bohonak, 1999; Kimura & Weiss, 1964; Slatkin, 1987; Wright, 1943, 1951). This body of theory predicts that species with weaker dispersal abilities or proclivities will exhibit greater genetic differentiation at smaller spatial scales due to reduced gene flow. The inverse expectation is a well‐mixed gene pool with weak genetic structuring, or even panmixia, for species with greater vagility. However, the effects of dispersal ability on spatial genetic structure have only been directly evaluated in a few study systems, including wind‐ versus animal‐dispersed seeds in plants (Hamrick et al., 1993), free‐swimming versus pelagic marine invertebrates (Chust et al., 2016), amphibians with different means of locomotion (Richardson, 2012), a community of small mammals with variable swimming abilities (Brunke et al., 2019), and even three lizard and one bird species in an urbanizing landscape in southern California, USA (Delaney et al., 2010). Animals that fly generally have lower levels of genetic structure across space due to an increased potential for gene flow at larger geographic distances (reviewed in Medina et al., 2018). However, flying and nonflying species have rarely been compared directly in the same landscape (Delaney et al., 2010).

Research into the evolutionary impacts of urban environments is still relatively new, but there is a rapidly growing body of literature to draw predictions from. Reviews of the literature have found that urbanization can impact gene flow, drift, and natural selection pressures, all potentially impacting genetic structure (Alberti et al., 2017; Johnson & Munshi‐South, 2017; Miles et al., 2019). However, these studies have also identified substantial variation in how, and the degree to which, urban habitat impacts these evolutionary processes. For example, Miles et al. (2019) found that 66% of studies examining nonadaptive urban evolution documented greater genetic differentiation in urban populations than paired nonurban populations, as would be expected if urbanization reduces connectivity and gene flow through fragmentation. However, 19% of the studies found evidence of reduced genetic differentiation in urban areas, suggesting that connectivity can sometimes increase in urban areas, likely through facilitation of movement in some species (Miles et al., 2019). In the same review, nearly a third of studies found higher genetic diversity within urban populations. This variation is due in part to the large range of study systems incorporated (i.e., specific cities and species investigated). An approach comparing multiple species within the same landscape is needed to move beyond this inherent variation and isolate how specific aspects of species ecology and urban environments shape important evolutionary processes, including gene flow (Richardson et al., 2016).

In this study, we directly compare spatial patterns of gene flow across the Providence, Rhode Island metropolitan region between white‐footed mice (*P. leucopus*) and big brown bats (*E. fuscus*), two native synanthropic species with very different modes of dispersal. To generate hypotheses about how urbanization will impact each species' movement and connectivity, we use the three models described earlier—which we will refer to as fragmentation, facilitation, and neutral hereafter. Consistent with the fragmentation model, we predicted that the lower dispersal capacity of white‐footed mice (~500 meters average dispersal distance, Stickel, 1968) would lead to more restricted movements, greater genetic differentiation, higher inbreeding levels, and lower genetic diversity, particularly in more urban parts of the study region. In contrast, big brown bats have daily foraging ranges three times the average dispersal distance of mice (Brigham, 1991). The facilitation model could be possible for bats if urban areas promote more movement than "natural" habitats. This would manifest as higher gene flow, lower genetic differentiation, and lower inbreeding within urban areas compared with exurban habitats, or if human transport led to the movement of animals (e.g., Miles et al., 2018), which is an unlikely scenario for bats. Hence, we predicted that bat movement would be less impacted by the urban landscape matrix, exhibiting lower levels of genetic differentiation and inbreeding across the study area, and higher genetic diversity. Our bat predictions are consistent with a null neutral model where urban landscapes have little effect on the connectivity of bats across the study region. This study represents one of the first to directly compare multiple species within the same landscape spanning a regional urban‐to‐rural landscape, an important gap to fill for urban ecology and evolution. Moreover, here we document the evolutionary impacts of dispersal capacity for native species that have persisted within a rapidly expanding urban environment.

## 2 METHODS

### 2.1 Study area and sampling

**FIGURE 1:**
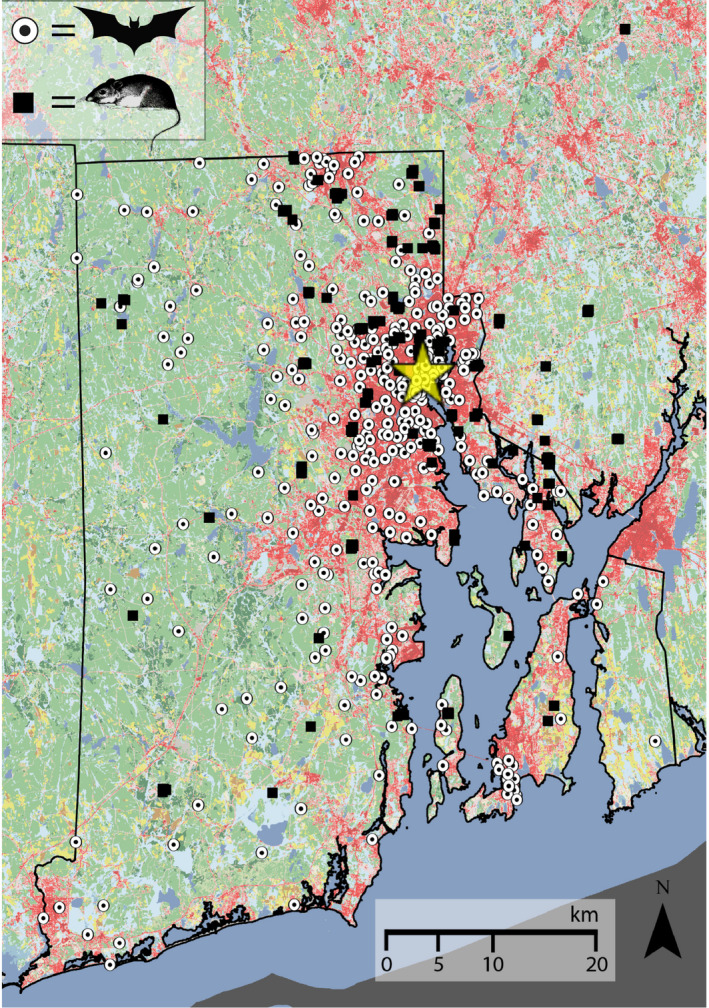
Land cover map of the focal study region in Rhode Island and southeast Massachusetts (~3,100 km^2^). *Peromyscus leucopus* mouse collection locations are represented with black squares, and white circled points denote *Eptesicus fuscus* bat sampling sites. Land cover data follow the standard U.S. Geological Service National Land Cover Database color scheme: red and pink pixels (30 × 30 meter resolution) represent developed areas of impervious surface; yellow pixels are crops; light and dark green is forest cover; blue pixels are open water. Narragansett Bay is the large body of water in the southeastern quadrant of the map. The yellow star indicates the urban core of the city of Providence

We collected tissue samples from both white‐footed mice (*P. leucopus*) and big brown bats (*E. fuscus*) between 2014 and 2019 across the Providence metropolitan area. This area encompasses all of Rhode Island, parts of southeastern Massachusetts, and is inhabited by approximately 1,600,000 people. This focal landscape includes ~3,500 km^2^ and is roughly bisected by the city of Providence (population size of 180,000) and the ~400 km^2^ Narragansett Bay (Figure 1). Both species are common and widely distributed across the study area. For the mice, 225 samples were collected by live trapping with Sherman live traps. We set traps in the afternoon and returned the following morning to collect 1 cm of tail tip tissue, after anesthetizing the tail with lidocaine. We then applied povidone–iodine to the tail and released the mouse. The tail tissue was placed in 95% ethanol and stored at −80°C once we returned to the laboratory. We obtained tissue samples from another 45 mice from the Harvard Museum of Comparative Zoology collections; an initial principal component analysis found no evidence that the museum specimens differed systematically from the samples we collected (Figure S1).

We collected bats as part of the Rhode Island Department of Health's (RI DOH) Rabies Surveillance Program. Nuisance bats found inside human‐occupied buildings were sent to the RI DOH testing facility, and we obtained tongue and ear tissue from 380 rabies‐negative specimens. Because this is a state monitoring program, these samples are only from the state of Rhode Island, and do not cross into Massachusetts (Figure 1).

### 2.2 DNA extraction and sequencing

DNA was extracted from mouse tail and bat tongue tissue using standard kit‐based extraction protocols (Qiagen DNeasy Blood and Tissue Kit) and included an RNase treatment step to maximize sequencing of DNA over RNA. For mice, we performed double‐digest restriction site‐associated DNA sequencing (ddRADseq) library preparation following the Peterson et al. (2012) protocol. In brief, DNA was restriction‐digested with the MluCI and SphI enzymes (New England Biolabs), followed by ligation of a P1 adapter that contained one of 48 unique five‐nucleotide barcodes, and a P2 adapter ligated to fragment overhangs. Once barcoded, samples were pooled into sets of 48, with equimolar concentrations of each sample within each pool. Fragments of 340–412 base pairs (bp) were isolated using a Pippin Prep (Sage Science) with 2% agarose cassette. We quantified the DNA concentrations of each pool using a Qubit fluorometer, followed by PCR amplification for 11 cycles using Phusion high‐fidelity reagents (New England Biolabs). The PCR also added Illumina flow cell annealing sequences and a second pool‐specific indexing barcode, giving each sample a unique dual barcode combination for downstream identification. Materials were bead‐cleaned using homemade Sera‐Mag magnetic beads (Fisher Scientific; Rohland & Reich, 2012) after each of the aforementioned steps. A final quality control step was done with an Agilent 2100 BioAnalyzer, and then, pooled libraries were sent to the New York Genome Center for 125‐bp paired‐end sequencing on an Illumina HiSeq 2500 instrument.

For bats, we performed quaddRAD library preparation following the protocol from Franchini et al. (2017) with modifications. The quaddRAD method includes adapters with four random nucleotides, which allows for removal of duplicate sequences generated during PCR amplification. Double restriction enzyme digestions were performed in 30 µl reactions using 15 U of the EcoRI‐HF and SphI‐HF restriction enzymes (New England Biolabs) and a minimum of 400 ng of genomic DNA. Obtained fragments were ligated with uniquely barcoded adaptor pairs (I5 and I7, Illumina) using 400 U of T4 DNA ligase (New England Biolabs) in 40 µl reactions. Individual PCRs with unique primer pairs (i5xx and i7xx, Illumina) were amplified in 14 PCR cycles using the Kapa Library Amplification Master Mix (Roche), according to their instructions. PCR products were quantified using Accublue dsDNA broad‐range kit (Biotium) and were pooled equimolarly in two library pools consisting of 193 and 195 samples, respectively. We also included in total eight replicate samples to calculate sequencing error within libraries. After pooling, both libraries were quantified using Quant‐it dsDNA broad‐range Assay kits (Invitrogen) and bead‐cleaned using homemade Sera‐Mag beads. Fragments of 373–455 bp were selected using a 2% ethidium free gel cassette on a BluePippin (Sage Science), and run on an Agilent 2100 BioAnalyzer (Applied Biosystems) to verify the distribution of fragments. Both libraries were sent to Admera Health for 150 bp paired‐end sequencing on an Illumina HiSeq 4000.

### 2.3 Sequencing data quality, variant calling, and filtering

For mice, which have a reference genome available from the congener *P. maniculatus*, we used the *process_radtags* script from Stacks (Catchen et al., 2013) to assign reads to individuals, followed by aligning reads to the Pman_2.1 reference genome using Bowtie2 with default settings (Langmead & Salzberg, 2012). We then ran the *ref_map* and *populations* pipeline, retaining only loci found in at least 90% of samples, (*r* = 0.90), with a minor allele frequency of at least 5% (min_maf = 0.05), and a heterozygosity upper bound of 0.8 (max_het = 0.8) in order to limit the effects of duplication within the genome. We also only retained one single nucleotide polymorphism (SNP) per RADtag (‐‐write_single_snp), to meet the assumptions of linkage equilibrium in subsequent analyses (e.g., PCA and fastStructure). For both species, we also created a dataset of 5,000 random SNPs using the whitelist function in *populations* within Stacks. This random subset of 5,000 SNPs was used in MEMGENE, while a bed2diffs file was generated for other analyses (i.e., sGD, see below). To investigate potentially sex‐biased dispersal patterns, we also ran populations with grouping samples by sex (using a popmap file) for both species.

For bats, we first used the *clone filter* and *process_radtags* modules in the Stacks 2.5 pipeline (Catchen et al., 2013; Rochette et al., 2019) to remove duplicate sequences, demultiplex sequenced data, and filter out poor‐quality reads, for each individual based on barcode information. Options to clean data (‐*c*), rescue barcodes (‐*r*), and discard reads with low‐quality scores (‐*q*) were applied with default values. Sequence quality assessment was performed using the software FastQC (http://www.bioinformatics.babraham.ac.uk/projects/fastqc/). Then, we removed 13 samples (out of 388) with a low number of reads (less than 500,000 paired‐end reads). Next, we employed the *denovo_map* pipeline in Stacks. Initially, we tested the assembly parameters and genotyping error by running the pipeline on a subset of samples including replicates. Values for the three main parameters −*m* (minimum number of identical raw reads to create a stack), −*M* (number of mismatches allowed between two alleles of a heterozygote), and −*n* (the number of mismatches between any two alleles) were chosen following the optimization procedure described by Rochette and Catchen (2017). Briefly, −*m* was set to 3, and increasing values for −*M* and −*n* were tested. We also used the replicated sample dataset to estimate genotypic errors by performing a relatedness analysis (identity‐by‐state), on SNP genotypes in the R package SNPrelate (Zheng et al., 2012). After this initial testing, we removed replicates (eight samples) and then reran the denovo_map pipeline with *M* = *n* = 4. We also calculated the number of missing SNPs per individual using VCFtools (Danecek et al., 2011) and kept all individuals with less than 25% of missing data. The final dataset consisted of 367 samples. All parameters for the Stacks population pipeline were identical to the mice (see above).

We tested for potentially adaptive loci in both species using the R package *pcadapt* (Luu et al., 2017) and default Mahalanobis distance method using the online protocol found at https://bcm‐uga.github.io/pcadapt/articles/pcadapt.html. The number of principal components was selected using the scree plot method, and the number of outliers was determined by identifying loci with a *qval* less than an alpha of 0.1, which equates to a false discovery rate less than 10%. We used a conservative false discovery rate to ensure the SNPs retained were neutral, and also removed all SNPs on nonautosomal sex chromosomes.

### 2.4 Spatially explicit mapping of genetic structure

The use of pairwise genetic distance estimates has faced increasing criticism for studies investigating spatial genetic structure, in part because pairwise data are not independent of one another. In addition, many species—including the mice and bats studied here—are continuously distributed across the landscape rather than isolated in discrete populations. For that reason, we attempted to sample individuals as uniformly as possible across the study region, with a focus in the urbanized portion of the landscape (Figure 1). We then analyzed the genomic data for these species with spatially explicit approaches designed to take location into account in identifying fine‐scale genetic structure, and also bypass the issues with analyses relying on pairwise genetic distances.

We performed a Moran's eigenvector mapping (MEM) analysis in the R package MEMGENE (Galpern et al., 2014). MEM creates new orthogonal, and spatially independent, variables that summarize the spatial relationship of genetic variation among sampled individuals (Peres‐Neto & Galpern, 2015). The proportion of shared alleles was used as the measure of genetic distance between individuals within each dataset (i.e., mice and then bats). We extracted the new MEM eigenvector variables from the analysis and visualized the first two axes of variation by mapping them onto their coordinates, hence producing a spatially explicit representation of genetic variation among samples.

We also used the sGD package in R to understand the distribution of genetic diversity across the study area (Shirk & Cushman, 2011). We calculated the inbreeding coefficient (*F_IS_*) and observed heterozygosity (H_obs_) for an expected genetic neighborhood surrounding each sample, with a radius of 10 km. We then visualized the output values using QGIS to plot on a map of our focal landscape.

Lastly, we visualized the estimated effective migration surfaces using EEMS (Petkova et al., 2016). The EEMS method assumes a stepping‐stone model (Kimura & Weiss, 1964) and uses a grid to group samples with known locations to a particular deme, which is located at the grid intersections. Migration rates are then estimated for the cells created by the grid and visualized using RStudio commands described in the EEMS instruction manual. The variances for the proposal distributions of the migration and diversity parameters were optimized by targeting acceptance percentages between 20% and 30%, as recommended by the authors. The number of MCMC iterations was set to 10 million with a 1 million burn‐in and 9,999 thin iteration. Once the proposal variance values were optimized, we ran a range of deme values for the mouse (250, 500, 750, and 1,000) and bat (50, 100, 200, and 400) data. Convergence of the MCMC chain was visually checked by plotting the posterior traces using the *eems.plot* function in rEEMSplots. If convergence was not attained, additional MCMC iterations were run and convergence was visually rechecked. The multiple runs with different numbers of demes were averaged in RStudio.

### 2.5 Urbanization impacts on population genetic traits

In order to evaluate how the degree of urbanization is related to the genetic traits we measured, we used ArcGIS to calculate the proportion of urban land cover surrounding each sampled mouse and bat location. Using the USGS National Land Cover Database raster, we added a 500 m buffer around each sampling point and used the Tabulate Area tool to produce a table of the number of cells of each land cover class within each sample's buffer. We then calculated the percentage of cells within the buffer comprised of low‐, medium‐, and high‐intensity developed land cover. These land cover categories represent areas with 20%–100% impervious surface within each 30 × 30 m cell, and are commonly used to quantify the degree of urbanization.

We conducted a linear regression between this metric of urbanization and the inbreeding coefficient produced in sGD (described above) using the "lm" function in base R. We then used the package *ggplot2* to plot this relationship (https://ggplot2.tidyverse.org/), denoting samples from the mainland versus islands to visually inspect for any differences in samples from these contiguous or insular landscape contexts. Lastly, we also performed this same analysis between urbanization and observed heterozygosity, a measure of genetic diversity.

### 2.6 Characterizing genetic clustering using multivariate and Bayesian approaches

The genetic clustering in mice and bats across our study region was assessed with a principal component analysis (PCA) using the function *glPCA()* implemented in the R package *adegenet* 2.1.2 (Jombart et al., 2008) in R. While PCA is most useful as an exploratory analysis and has limited power to pull out clusters, we were most interested in looking for any differences in the genetic structure of males versus females separately for both species. Both species exhibit male‐biased dispersal (Mossman & Waser, 1999; Arnold, 2007), meaning that males can be expected to move more frequently and farther. This would result in females showing more genetic structure than males. Plots were generated using *ggplot2* (Wickham, 2009). We also used a variational Bayesian framework for calculating posterior distributions in fastStructure (Raj et al., 2014), with default options (using both the *simple* and *logistic* prior), to assess the number of genetic clusters (*K*) without any prior information regarding population identity or geographic location for both species. The upper *K* limit was set to *K* = 20 and the *chooseK.py* function was used in order to select the appropriate number of model components that explain structure in each dataset.

## 3 RESULTS

### 3.1 SNP dataset and summary statistics

We obtained 206,139 and 26,736 SNPs for 215 mice and 367 bats, respectively, postfiltering (Table S1). We identified 16,622 outlier SNPs (7.5%) for the mice and 810 outlier SNPs (2.5%) for the bats. Thus, the mice had more than three times the number of potentially adaptive SNPs compared with the bats. There was a similar genetic variation (e.g., observed and expected heterozygosity) between the two species with the exception of the inbreeding coefficient being higher in bats (Table 1).

**TABLE 1:** Summary of genetic diversity indices in white‐footed mice (*Peromyscus leucopus*) and big brown bats (*Eptesicus fuscus*). The number of all polymorphic sites (N_sites_), observed and expected heterozygosity (H_obs_, H_exp_), nucleotide diversity (Pi) and inbreeding coefficient (*F_IS_*). Standard error (StdErr) values are shown in parentheses

Species	N_sites_	H_obs_ (StdErr)	H_exp_ (StdErr)	Pi (StdErr)	*F_IS_* (StdErr)
Big brown bats	26,736	0.212 (0.00065)	0.251 (0.00072)	0.251 (0.00072)	0.15 (0.056)
White‐footed mice	206,139	0.228 (0.00026)	0.254 (0.00028)	0.255 (0.00028)	0.09 (0.014)

### 3.2 Spatially explicit mapping of genetic structure

**FIGURE 2:**
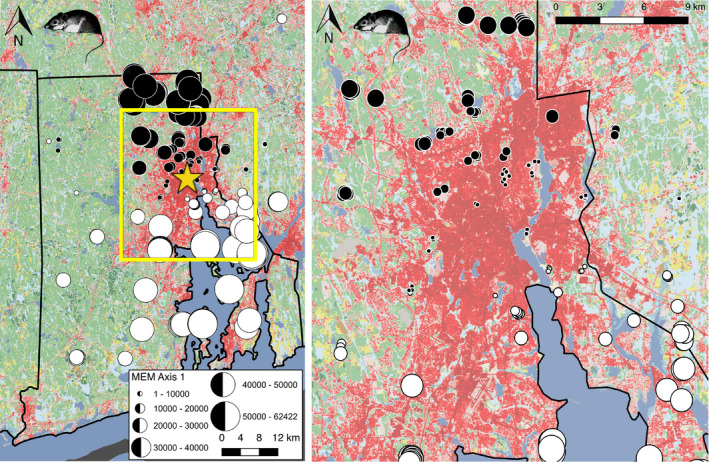
(a) Moran's eigenvector mapping (MEM) shows divergence between mice within and north of Providence (yellow star) and Narragansett Bay (the large body of water in southeast quadrant of map). Circle color and size representing genetic similarity along the first MEM variable axis (explaining 9.1% of overall genomic variation in SNP data). (b) Inset area within the yellow box of (a), highlighting the sharp genetic transition within the city of Providence

Moran's eigenvector mapping (MEM) analysis, which looks at genetic variation after accounting for patterns of spatial autocorrelation, found a sharp shift in the genetic signature of mice along a south‐to‐north axis of the study region (Figure 2a), with a conspicuous break between mice in the urban core of Providence (Figure 2b). Only two mice—one at the Durfee Management Area (at the western edge of our sampling) and one in Sharon, Massachusetts (at the far northeastern edge of sampling)—deviated from this pattern across the entire 215 mouse dataset. For mice, 14% of the genetic variation was explained by the first two MEM eigenvector axes. In stark contrast, the MEM analysis for bats found no significant eigenvectors, meaning there was no detectable structure in the genomic data. The lack of eigenvectors prevented us from plotting these data since no axis of variation was generated.

**FIGURE 3:**
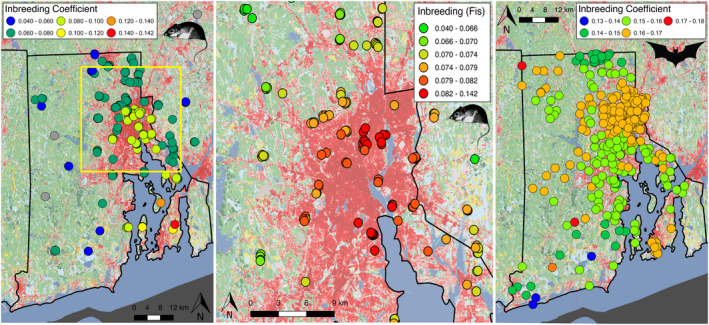
The inbreeding coefficient (*F_IS_*) for each sampled (a) mouse, and (c) bat across the study landscape. (b) The same mouse inbreeding coefficient distribution as in (a), but zoomed into the urban and peri‐urban areas around Providence, Rhode Island (population ~1,000,000 people), showing higher *F_IS_* around this urban core. The gray points are samples that did not meet the sGD minimum criteria of 5 samples within the 10 km neighborhood radius (*min_N* = 5 parameter setting)

Spatially explicit analyses of genetic diversity based on overlapping genetic neighborhoods in sGD found spatial heterogeneity in both levels of inbreeding and heterozygosity. Inbreeding coefficients (*F_IS_*) in mice were highest on the three islands, and in the urbanized core of Providence and surrounding developed landscape (Figure 3a,b). *F_IS_* was generally similar for most bats in the study area, and was not consistently associated with islands or urban habitat (Figure 3c). Bats generally had higher *F_IS_* values in this dataset than mice, though they did not vary consistently across the region (Figure 3c). Genetic diversity (observed heterozygosity) was reduced only for the mice sampled on islands, with no signal of reduced heterozygosity in urban parts of the matrix (Figure S3a). There was no clear signal of heterozygosity varying in relation to islands or urban habitat for bats (Figure S3b).

**FIGURE 4:**
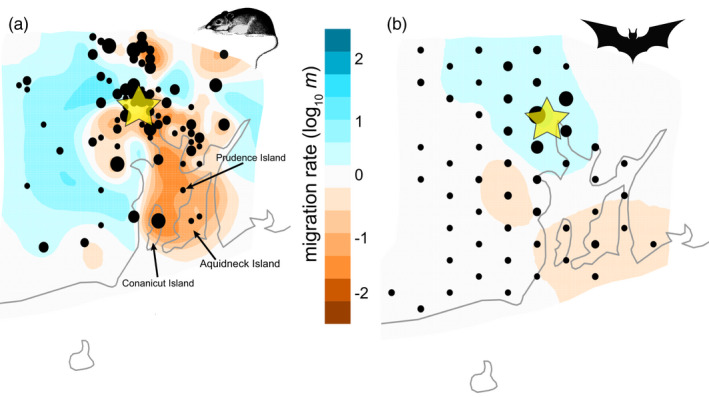
Estimated effective migration surface (EEMS) visualizations for (a) white‐footed mice (*n* = 215) and (b) big brown bats (*n* = 367). The maps represent the average of multiple EEMS runs with different numbers of demes. Points that are located near a given vertex created by the deme grid are aggregated, which is why the sample locations are not the actual sample coordinates. Yellow star denotes the location of the Providence urban core

The visualization of effective migration rates (EEMS) also showed a starkly different pattern between the mice and bats. The mice showed restricted migration through the Providence urban core, as well as in the urbanized areas of Woonsocket/Cumberland/Lincoln (population ~100,000) and between the island and mainland mice (Figure 4a). The bats showed much less variation in effective migration relative to the mice, indicating no sharp barriers exist that limit bat connectivity (Figure 4b). Bats did exhibit slightly higher migration than expected under an isolation‐by‐distance scenario through the urbanized areas of the Providence area and northwest. Both the mice and bats showed restricted effective migration toward the islands, but to varying degrees (Figure 4).

### 3.3 Urbanization impacts on population genetic traits

**FIGURE 5:**
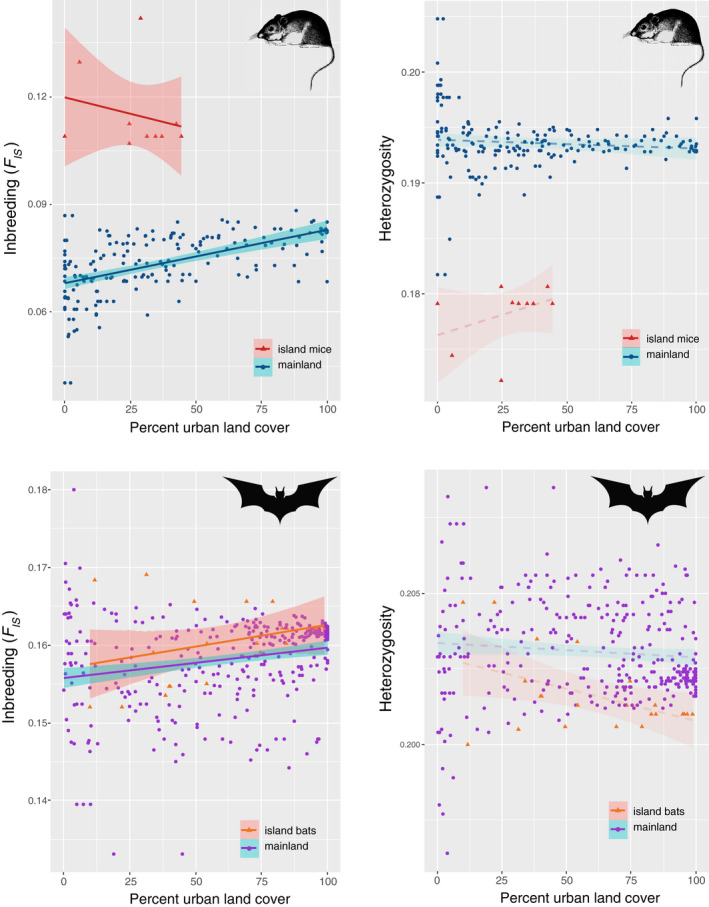
Linear regression analyses examining the association between urbanization (the percentage of urbanized land cover within 500 meters of each sample) and the level of inbreeding (*F_IS_* inbreeding coefficient, panel a & c) or observed heterozygosity (b & d) in mice and bats. The inbreeding level for both species was significantly positively associated with urbanization (*p* < .0001), denoted with solid trend lines and confidence intervals. Heterozygosity was not associated with urbanization in either the mice (*p* = .786) or bats (*p* = .079), denoted with dashed trend lines and translucent confidence intervals

The percent urban land cover within 500 m of each sampled animal ranged from zero to 100% within the study region. For mice, there was a significant positive relationship between urbanization and inbreeding (*F_IS_*, *p* < .0001, *r* ^2^ = 0.102), and island mice exhibited uniformly higher *F_IS_* levels (Figure 5a). Though weaker, this same pattern was observed for bats (Figure 5c, *p* < .001, *r* ^2^ = 0.051). Observed heterozygosity—a metric of genetic diversity—was not associated with urban land cover for either species (Figure 5b,d; *p*_mouse_ = .786, *r* ^2^_mouse_ = 0.0004; *p*_bat_ = .080, *r* ^2^_bat_ = 0.009).

### 3.4 Characterizing genetic clustering using multivariate and Bayesian approaches

The total variance explained in the first two axes of the PCAs was generally low. There was greater variation in the first two axes of the mouse dataset (average of 3.41% between males and females) than in the bats (1.97% total). PCA provided no clear signal of land cover; however, female bats showed more structuring (2.57% total variance) than males (1.33% total variance; Figure S4). There was no apparent difference among sexes for mice (Figure S4). The fastSTRUCTURE analyses showed that the most appropriate number of model components that maximizes marginal likelihood is K = 1 for both species (i.e., no structure). When forced beyond K = 1, samples from rural sites (e.g., Jamestown, Bristol, Warren) show substructure at K = 4 for the mice and at K = 9 for the bats (Figure S2).

## 4 DISCUSSION

Ecologists and evolutionary biologists have long been focused on the role that dispersal plays in the dynamics of populations. Dispersal leading to gene flow can bolster population survival through rescue effects, or restrict local adaptation via migration load (Gonzalez et al., 2013; Hufbauer et al., 2015; Lenormand, 2002). While much theory has been developed around dispersal impacts on populations, few empirical studies compare species with differing dispersal abilities in the same focal landscape, particularly urban landscapes. In this study, we directly compared white‐footed mice to big brown bats. Both are widely distributed synanthropic species, but with very different movement abilities, allowing us to explore how dispersal and urbanization interact to fragment, facilitate, or have limited impact on the connectivity of these species. Our results show clear differences between these species in their patterns of genetic structure that are consistent with those movement capacities. For *Peromyscus* mice, levels of inbreeding were elevated within the highly urbanized landscape in and around Providence, Rhode Island, as well as on the three islands that were sampled. In addition, there was a sharp genetic transition zone running through the urban core of Providence. These data support the fragmentation model of urbanization restricting the connectivity of mice across the study region. In contrast to mice, bats showed very little signal of genetic structuring across the entire study area using spatially explicit methods. This finding suggests that bats in the study region represent a near‐panmictic gene pool, likely due to volant dispersal abilities, and supports the neutral model of connectivity where bats are largely unaffected by urbanization. There were, however, weak signals of lower migration to the islands and elevated inbreeding as urbanization increased for bats.

### 4.1 How dispersal ability and urbanization shape genetic structure

White‐footed mice rarely disperse more than 500 meters (Stickel, 1968), with home ranges limited to around 1,000 m^2^ (Teferi & Millar, 1994), and likely much less in urban patches. Consistent with this limited dispersal, we found clear genomic structuring in *P. leucopus* mice with spatially explicit MEM analyses, notably along a 60 km south‐to‐north axis (Figure 2a). The sharpest transition along this axis occurred within the urbanized core of Providence (Figure 2b). A similar pattern was seen for effective migration with EEMS, with lower migration rates through the urban core of Providence, as well as in other urban areas like the Woonsocket/Cumberland/Lincoln developed corridor in the northern part of the study region (Figure 4a). Mice also showed a larger signal of genetic divergence than bats between mainland and island populations, both in the EEMS and in the *F_IS_* analyses (Figures 3a, 4a and 5a).

Our results are consistent with previous studies on the effects of urban habitat on synanthropic mice. Genetic data indicate that *P. leucopus* in New York City are isolated within city green spaces, experiencing little gene flow among the populations and movement heavily reliant on vegetation cover (Munshi‐South, 2012; Munshi‐South & Kharchenko, 2010). Like our study, another project on NYC mice expanded the focal region to include an urban‐to‐rural gradient outside of the city, and found an inverse relationship between urban land cover and several metrics of genetic diversity (Munshi‐South et al., 2016), indicating that urbanization reduces gene flow. These restrictions in gene flow may promote local adaptation in *Peromyscus* to selection imposed by urban food resources and soil contamination (Harris & Munshi‐South, 2017; Harris et al., 2013). Studies on ecologically similar *Apodemus* mouse species in Europe have also found increased genetic differentiation and relatedness, and lower genetic diversity for urban populations (Gortat et al., 2013, 2017). Other species with modest dispersal abilities show similar patterns to mice in our study. For example, Fusco et al. (2021) found increased genetic differentiation among urban and suburban salamander populations, compared with rural locations. Consistent with the mice and bats in the current study, they also found no association of urbanization with a heterozygosity metric of genetic diversity (Fusco et al., 2021).

In stark contrast to the mice, big brown bats in our study region showed little signal of spatial genetic structure. The MEM analysis was unable to identify even a single significant eigenvector in the bat SNP data, and the EEMS estimates of effective migration showed minimal variation across the study region (Figure 4b). This supports our prediction that gene flow would be much higher in the volant bat than in a terrestrial rodent. However, we did not predict a signal of panmixia over the entire ~3,500 km^2^ study area. Rather than true panmixia (where all individuals in the population have an equal chance of mating with every other individual), the dispersal, life history, and social structure of *E. fuscus* likely means that their gene pool is well mixed enough at our studies' spatial scale to prevent substructuring over generations. The range of *F_IS_* values was much narrower for bats across the study region, with a less clear signal of spatial variation relative to the mice (Figure 3c). This lack of marked spatial genetic structure is consistent with other flying species. For example, Carlen and Munshi‐South (2021) found little evidence of genetic structure and high estimates of migration over hundreds of kilometers (Virginia to Connecticut, U.S.A.) in urban pigeons, while a study on house sparrows found minimal differentiation with coordinate analyses in response to urbanization at a much smaller scale (ca. 10 km; Vangestel et al., 2012). Bumblebees that span an urban‐to‐rural gradient also exhibited little genetic structure in response to urbanization, despite their small body size (Theodorou et al., 2018). At a much larger continental scale, a recent review and re‐analysis of available microsatellite data found that bird species (*n* = 7 nonmigratory species) generally have higher genetic diversity and less genetic differentiation than the mammal species evaluated (*n* = 41; Schmidt et al., 2020). These genetic differences increased as human population density increased, consistent with the process of dispersal mitigating genetic structuring with urbanization.

Through recapture data of the big brown bats in our study area, we have seen individual bats move up to 27 km during long‐distance dispersal events, and acoustic surveys detect pulses of activity and flight movements along the coast of Narragansett Bay in late summer (C. Brown et al., unpublished data). Previous studies have observed long‐distance dispersal in bats, particularly for males, which minimized genetic structure in those populations (Hua et al., 2013; Norquay et al., 2013). Turmelle et al. (2011) found very little spatial genetic variation for *E. fuscus* across their entire North American range using a small number of markers, and coalescent analysis suggested that the signal from mtDNA and nuclear markers resulted from increased gene flow in males. Razgour et al. (2014) found some structure related to foraging habitat connectivity in gray long‐eared bat (*Plecotus austriacus*), a species that is inefficient with long‐distance flight (Norberg & Rayner, 1987), but at the scale of the European continent. Studies comparing multiple bat species have also identified vagility as an important factor in genetic structuring (Campbell et al., 2006; Heaney et al., 2005; Rossiter et al., 2012). In addition, female *E. fuscus* exhibit higher levels of philopatry to natal roosts, further limiting gene flow relative to males (Arnold, 2007). This is consistent with what we saw with a post hoc principal component analysis performed for each sex of bats (Figure S4c,d), with no corresponding sex‐dependent signal in mice (Figure S4a,b).

Big brown bats are year‐round residents (i.e., nonmigratory) that roost and hibernate individually or in small groups within our study area, typically in hollow trees, attics, barns, and other structures. *Eptesicus fuscus* have a polygynous mating system, with reproductive stage males and females hibernating together and mating over the fall and winter. Females typically congregate in maternity colonies during the summer, which rarely include the more dispersive males. As with other bats, *E. fuscus* is long‐lived (Hitchcock et al., 1984). Together, these social, behavioral, and life history traits may contribute to the lack of any genetic structure. Comparing seven bat species within the same small landscape in Malaysia, Rossiter et al. (2012) found more genetic structuring in tree‐roosting species than species that roosted communally in cave colonies. A study on Bechstein's bats (*Myotis bechsteinii*) in Germany found some mtDNA, but little nuclear DNA structuring in this highly insular, colonial species (Kerth & Van Schaik, 2012). This again supports the general lack of population genetic structuring in many bat species and the potential differences between males and females.

Both species exhibited a significant association between inbreeding and urbanization, with *F_IS_* increasing as the urban land cover around each sample increased (Figure 5). The association was stronger for mice than bats, with variation in urbanization explaining twice the variation in *F_IS_* for mice relative to bats. Mice on islands showed uniformly higher *F_IS_* levels than mainland mice (Figure 5a), a pattern not shared by the bats (Figure 5c). Interestingly, the pattern of *F_IS_* versus percent urban land cover was the opposite for island and mainland mice. This could be because the impact of being on an island for mice is stronger than the effect of urbanization due to the limited ability of mice to move to or from islands, or the fact the urban patch “islands” have been around for much shorter periods of time relative to offshore islands. However, our sample sizes on islands were limited for mice and did not span the full range of percent urban land cover. Neither species showed any association between urbanization and heterozygosity, suggesting that genetic diversity has not declined in urban areas to date (Figure 5b,d).

### 4.2 Variable responses to urbanization

Urbanization and its proxies have been linked with impacts on gene flow that shape spatial genetic structure (Johnson & Munshi‐South, 2017). Additionally, a recent synthesis of available population genetic data found substantial variation across species in 167 studies evaluating how levels of gene flow and drift differ between urban and nonurban areas (Miles et al., 2019). In our study, mice in the urbanized areas within and around Providence had elevated inbreeding levels (Figure 2a,b). Mice sampled on islands also exhibited higher inbreeding, which is consistent with island populations sharing limited gene flow with mainland populations (Frankham, 1997). In some situations, highly isolated forest fragments surrounded by dense urbanization may provide habitat that is similar to islands (Munshi‐South & Kharchenko, 2010). However, the exact mechanism responsible for higher inbreeding within the urban core in our study is unclear. Restrictions in gene flow within highly developed landscapes may create insular gene pools or perhaps an altered social structure with smaller breeding groups. Roads have also been linked with genetic structuring in *P. leucopus* (Howell et al., 2017), as well as other rodents in cities (Combs et al., 2018; Richardson et al., 2017). Inbreeding for bats also showed an association with urbanization, although weaker and not clearly tied to the Providence urban core (Figures 3c and 5c). Previous studies comparing different bat species have found an effect of disturbed habitat or developed landscapes in reducing gene flow (Cleary et al., 2017; Heaney et al., 2005), though none have focused on urban landscapes.

While dispersal and the resulting gene flow do mix gene pools, other evolutionary factors can also lead to genetic divergence over time. Genetic drift is a random process leading to genetic divergence over time, and it increases in isolated or small populations. Adaptive divergence in response to local natural selection pressures occurs faster with restricted gene flow, accelerating genetic differentiation (Lenormand, 2002). Urban‐developed landscapes can create strong novel selection pressures, and even increase background mutation rates (Johnson & Munshi‐South, 2017; Yauk & Quinn, 1996). Patterns of genetic differentiation and substructuring over space are thus influenced by not only dispersal and gene flow, but also the mutation–drift–selection balance within those study populations (Walsh & Lynch, 2018; Yeaman & Otto, 2011). The data collected for this study cannot address these other evolutionary processes, but the increased levels of inbreeding we see for both species in more urbanized areas could result in a reduced ability to adapt to future environmental changes (Keller & Waller, 2002), or even an increased ability to adapt in systems subject to strong local selection pressures and outbreeding depression (Lenormand, 2002).

The principal component analyses showed no signal of urban collected mice or bats being genetically differentiated from exurban individuals (Figure S4). The PCA results may differ because the other methods described earlier (e.g., MEM, *F_IS_* mapping) are spatially explicit, meaning they account for spatial locations, autocorrelation, and potential genetic neighborhoods to provide a more sophisticated picture of fine‐scale genetic structure (Galpern et al., 2014; Shirk & Cushman, 2011). The sGD analyses also are designed for continuous populations, unlike other patch‐based analyses that are based on an island model (Wright, 1931) of population structure (Shirk & Cushman, 2011). The fastStructure Bayesian clustering analysis suggested a single genetic unit unless using nonlogistic parameters, in which it showed sharp genetic clustering. However, few of the clusters corresponded to any known geographic variation across the study area. Based on past simulation research, the spatially explicit analyses presented earlier should be more powerful in looking at spatial genetic structure (Galpern et al., 2014; Petkova et al., 2016; Shirk & Cushman, 2011).

### 4.3 Evidence for fragmentation, facilitation, and neutral models

Most research looking into how urbanization impacts the connectivity of wild populations has focused on urban land cover as a fragmenting force that reduces gene flow. While this is typically the case (ca. 66% of studies; Miles et al., 2019), there is a growing appreciation of other species whose movement does not appear to be impacted at all by urban habitats (ca. 16%), or whose movement is even facilitated by urban habitats, or the people moving within and among cities (ca. 19%; Miles et al., 2019). We used these three mechanisms that potentially shape gene flow to guide our predictions. Our prediction that urbanization would fragment mice with limited dispersal abilities was supported by the sharp genetic breaks (Figure 2a) and higher *F_IS_* (Figure 3a,b) in and around the Providence urban core, as well as by the areas of very low migration (Figure 4a). We also predicted that bats would be less likely to suffer from fragmentation or reduced gene flow because of their ability to fly and extensive use of all parts of the urban core habitat. We observed these patterns, but were surprised at just how little genetic structure there was, particularly in the MEM and EEMS analyses. This finding is more consistent with the neutral, null model that urbanization has little effect on bats than the facilitation model, because there was no evidence of higher gene flow within urban habitats as compared to exurban areas. The data suggest that bats can readily move around regardless of land cover, though there is a weak signal that movements are reduced over water to islands. It is possible that the facilitation model is relevant for bats, but given their general ability to move and spatial genetics suggesting panmixia, it may just be difficult to pull out any signal of bats moving at higher rates through urban land cover. The secondary mechanism of human transport of animals facilitating gene flow through and among cities (e.g., Miles et al., 2018) seems unlikely for bats, given their behavioral preference for high roosting sites.

### 4.4 Conclusions and insights into native species

We found that gene flow in big brown bats is less impacted by urbanization than that of white‐footed mice along the same urban‐to‐rural gradient. This supports the theoretical expectation that species with greater dispersal capacity will maintain higher levels of connectivity, even as their habitat becomes fragmented during landscape development. More work in other cities will need to be done before we know how representative our data are for other locations. But the Providence metropolitan area certainly represents a large urban‐to‐rural landscape matrix, with 1,600,000 people total, and a population density of 3,736 people per km^2^ in the urban core. Understanding these patterns and their underlying mechanisms is important for urban ecology (e.g., occupancy/persistence) and evolutionary biology (drift–mutation–selection dynamics), particularly investigating multiple species in the same landscape. In addition, urbanization is a form of habitat fragmentation for many native species, and the residual patches surrounded by less suitable urban habitat can sometimes operate like true islands (Olejniczak et al., 2018). While not the main focus of this study, we do have direct comparisons between urban fragments in Providence and the islands we sampled in Narragansett Bay. Both species showed evidence of more restricted gene flow between islands and mainland populations relative to urban and rural comparisons, regardless of the overall level of genetic structure. This makes sense, as true islands (and their populations of bats and mice) have been “fragmented” from the mainland for longer than the urban fragmentation of Providence over the last century or two.

White‐footed mice in our study exhibited higher levels of inbreeding within the urbanized landscape around Providence. Our trapping efforts indicate that capture probabilities were much higher within or adjacent to patches of trees, often near parks and cemeteries. Therefore, the movement of mice likely relies on some level of connectivity between vegetated areas, making dispersal harder into and out of these urbanized islands of green space (Munshi‐South, 2012). Therefore, the long‐term persistence of *Peromyscus* mice in urbanizing landscapes likely depends on the maintenance or creation of patches of secondary urban forests and canopy cover. Native big brown bats, on the other hand, showed minimal evidence of restricted gene flow. This suggests that *E. fuscus* across the region are well connected via flight, maintaining levels of genetic variation and perhaps able to supplement local populations if they decline in the future. Big brown bats are no doubt aided in their synanthropy by their rapid colonization and adaptable use of urban infrastructure, such as buildings and bridges (Neubaum et al., 2007). Their habitat selection is even tied to socioeconomic indicators within cities (Li et al., 2019). While there is no evidence that the dispersal of *E. fuscus* is restricted within the city of Providence, or even the entire ~3,100 km^2^ area of Rhode Island, tree‐roosting bats require specific microhabitats with roosting sites and prey resources (Grindal & Brigham, 1999). Therefore, big brown bats can be sensitive to changes in habitat quality at small spatial scales despite panmictic population genetic patterns.

## CONFLICT OF INTEREST

None declared.

## Supporting information

Supplementary Material

## Data Availability

Data for this study will be available at the Dryad Digital Repository after the manuscript is accepted for publication.

## References

[eva13133-bib-0001] Abedi‐Lartey, M. , Dechmann, D. K. N. , Wikelski, M. , Scharf, A. K. , & Fahr, J. (2016). Long‐distance seed dispersal by straw‐coloured fruit bats varies by season and landscape. Global Ecology and Conservation, 7, 12–24. 10.1016/j.gecco.2016.03.005

[eva13133-bib-0002] Alberti, M. , Correa, C. , Marzluff, J. M. , Hendry, A. P. , Palkovacs, E. P. , Gotanda, K. M. , Hunt, V. M. , Apgar, T. M. , & Zhou, Y. (2017). Global urban signatures of phenotypic change in animal and plant populations. Proceedings of the National Academy of Sciences of the United States of America, 114, 8951–8956. 10.1073/pnas.1606034114 28049817 PMC5576774

[eva13133-bib-0003] Arnold, B. D. (2007). Population structure and sex‐biased dispersal in the forest dwelling vespertilionid bat, *Myotis septentrionalis* . The American Midland Naturalist, 157(2), 374–384. 10.1674/0003-0031(2007)157[374:PSASDI]2.0.CO;2

[eva13133-bib-0004] Barko, V. A. , Feldhamer, G. A. , Nicholson, M. C. , & Davie, D. K. (2003) Urban habitat: A determinant of white‐footed mouse (*Peromyscus leucopus*) abundance in Southern Illinois. Southeastern Naturalist, 2, 369–376. 10.1656/1528-7092(2003)002[0369:UHADOW]2.0.CO;2

[eva13133-bib-0005] Bierwagen, B. G. (2007). Connectivity in urbanizing landscapes: The importance of habitat configuration, urban area size, and dispersal. Urban Ecosystems, 10(1), 29–42. 10.1007/s11252-006-0011-6

[eva13133-bib-1001] Bohonak, A. J. (1999). Dispersal, gene flow, and population structure. Quarterly Review of Biology, 74(1), 21–45. 10.1086/392950 10081813

[eva13133-bib-0006] Brigham, R. M. (1991). Flexibility in foraging and roosting behaviour by the big brown bat (*Eptesicus fuscus*). Canadian Journal of Zoology, 96, 117–121.

[eva13133-bib-0007] Brunke, J. , Radespiel, U. , Russo, I. R. , Bruford, M. W. , & Goossens, B. (2019). Messing about on the river: The role of geographic barriers in shaping the genetic structure of Bornean small mammals in a fragmented landscape. Conservation Genetics, 20, 691–704. 10.1007/s10592-019-01159-3

[eva13133-bib-1002] Campbell, P. , Schneider, C. J. , Adnan, A. M. , Zubaid, A. , & Kunz, T. H. (2006). Comparative population structure of Cynopterus fruit bats in peninsular Malaysia and southern Thailand. Molecular Ecology, 15, 29–47. 16367828 10.1111/j.1365-294X.2005.02769.x

[eva13133-bib-0008] Carlen, E. , & Munshi‐South, J. (2021). Widespread genetic connectivity of feral pigeons across the Northeastern megacity. Evolutionary Applications, 14(1), 150–162. 10.1111/eva.12972. PMC7819573 33519962

[eva13133-bib-0009] Catchen, J. , Hohenlohe, P. A. , Bassham, S. , Amores, A. , & Cresko, W. A. (2013). Stacks: An analysis tool set for population genomics. Molecular Ecology, 22, 3124–3140. 10.1111/mec.12354 23701397 PMC3936987

[eva13133-bib-0010] Chust, G. , Villarino, E. , Chenuil, A. , Irigoien, X. , Bizsel, N. , Bode, A. , Broms, C. , Claus, S. , Fernández de Puelles, M. L. , Fonda‐Umani, S. , Hoarau, G. , Mazzocchi, M. G. , Mozetič, P. , Vandepitte, L. , Veríssimo, H. , Zervoudaki, S. , & Borja, A. (2016). Dispersal similarly shapes both population genetics and community patterns in the marine realm. Scientific Reports, 6, 1–12. 10.1038/srep28730 27344967 PMC4921837

[eva13133-bib-0011] Cleary, K. A. , Waits, L. P. , & Finegan, B. (2017). Comparative landscape genetics of two frugivorous bats in a biological corridor undergoing agricultural intensification. Molecular Ecology, 26, 4603–4617. 10.1111/mec.14230 28672105

[eva13133-bib-0012] Combs, M. , Byers, K. A. , Ghersi, B. M. , Blum, M. J. , Caccone, A. , Costa, F. , Himsworth, C. G. , Richardson, J. L. , & Munshi‐South, J. (2018). Urban rat races: Spatial population genomics of brown rats (*Rattus norvegicus*) compared across multiple cities. Proceedings of the Royal Society B‐Biological Sciences, 285, 20180245. 10.1098/rspb.2018.0245 PMC6015871 29875297

[eva13133-bib-0013] Danecek, P. , Auton, A. , Abecasis, G. , Albers, C. A. , Banks, E. , DePristo, M. A. , Handsaker, R. E. , Lunter, G. , Marth, G. T. , Sherry, S. T. , McVean, G. , & Durbin, R. (2011). The variant call format and VCFtools. Bioinformatics, 27(15), 2156–2158. 10.1093/bioinformatics/btr330 21653522 PMC3137218

[eva13133-bib-0014] Delaney, K. S. , Riley, S. P. D. , & Fisher, R. N. (2010). A rapid, strong, and convergent genetic response to urban habitat fragmentation in four divergent and widespread vertebrates. PLoS One, 5, e12767 10.1371/journal.pone.0012767 20862274 PMC2940822

[eva13133-bib-0015] Franchini, P. , Monné Parera, D. , Kautt, A. F. , & Meyer, A. (2017). quaddRAD: A new high‐multiplexing and PCR duplicate removal ddRAD protocol produces novel evolutionary insights in a nonradiating cichlid lineage. Molecular Ecology, 26(10), 2783–2795. 10.1111/mec.14077 28247584

[eva13133-bib-1003] Frankham, R. (1997). Do island populations have less genetic variation than mainland populations? Heredity, 78, 311–327. 9119706 10.1038/hdy.1997.46

[eva13133-bib-0016] Fusco, N. A. , Pehek, E. , & Munshi‐South, J. (2021). Urbanization reduces gene flow but not genetic diversity of stream salamander populations in the New York City metropolitan area. Evolutionary Applications, 14(1), 96–116. 10.1111/eva.13025. PMC7819553 33519959

[eva13133-bib-0017] Galpern, P. , Peres‐Neto, P. , Polfus, J. , & Manseau, M. (2014). MEMGENE: Spatial pattern detection in genetic distance data. Methods in Ecology and Evolution, 5, 1116–1120. 10.1111/2041-210X.12240

[eva13133-bib-0018] Gliwicz, J. , Goszczynski, J. , & Luniak, M. (1994). Characteristic features of animal populations under synurbization ‐ the case of the blackbird and of the striped field mouse. Memorabilia Zoologica, 49, 237–244.

[eva13133-bib-0019] Gomulkiewicz, R. , & Holt, R. D. (1995). When does evolution by natural selection prevent extinction. Evolution, 49, 201–207. 10.1111/j.1558-5646.1995.tb05971.x 28593677

[eva13133-bib-0020] Gonzalez, A. , Ronce, O. , Ferriere, R. , & Hochberg, M. E. (2013). Evolutionary rescue: An emerging focus at the intersection between ecology and evolution. Philosophical Transactions of the Royal Society B: Biological Sciences, 368(1610), 20120404 10.1098/rstb.2012.0404 PMC3538460 23209175

[eva13133-bib-0021] Gortat, T. , Rutkowski, R. , Gryczynska, A. , Kozakiewicz, A. , & Kozakiewicz, M. (2017). The spatial genetic structure of the yellow‐necked mouse in an urban environment – A recent invader vs. a closely related permanent inhabitant. Urban Ecosystems, 20, 581–594. 10.1007/s11252-016-0620-7

[eva13133-bib-0022] Gortat, T. , Rutkowski, R. , Gryczynska‐Siemiatkowska, A. , Kozakiewicz, A. , & Kozakiewicz, M. (2013). Genetic structure in urban and rural populations of *Apodemus agrarius* in Poland. Mammalian Biology, 78, 171–177. 10.1016/j.mambio.2012.07.155

[eva13133-bib-0023] Grindal, S. D. , & Brigham, R. M. (1999). Impacts of forest harvesting on habitat use by foraging insectivorous bats at different spatial scales. Ecoscience, 6, 25–34. 10.1080/11956860.1999.11952206

[eva13133-bib-0024] Gross, J. , Elvinger, F. , Hungerford, L. L. , & Gehrt, S. D. (2012). Raccoon use of the urban matrix in the Baltimore Metropolitan Area, Maryland. Urban Ecosystems, 15(3), 667–682. 10.1007/s11252-011-0218-z

[eva13133-bib-0025] Haldane, J. B. S. (1930). A mathematical theory of natural and artificial selection. (Part VI, Isolation.). Mathematical Proceedings of the Cambridge Philosophical Society, 26, 220–230. 10.1017/S0305004100015450

[eva13133-bib-0026] Hamrick, J. L. , Murawski, D. A. , & Nason, J. D. (1993). The influence of seed dispersal mechanisms on the genetic structure of tropical tree populations. Vegetatio, 107, 281–297.

[eva13133-bib-0027] Harris, S. E. , & Munshi‐South, J. (2017). Signatures of positive selection and local adaptation to urbanization in white‐footed mice (*Peromyscus leucopus*). Molecular Ecology, 26, 6336–6350. 28980357 10.1111/mec.14369 PMC5716853

[eva13133-bib-0028] Harris, S. E. , Munshi‐South, J. , Obergfell, C. , & O'Neill, R. (2013). Signatures of rapid evolution in urban and rural transcriptomes of white‐footed mice (*Peromyscus leucopus*) in the New York metropolitan area. PLoS One, 8, e74938 10.1371/journal.pone.0074938 24015321 PMC3756007

[eva13133-bib-0029] Heaney, L. R. , Walsh, J. S. , & Peterson, A. T. (2005). The roles of geological history and colonization abilities in genetic differentiation between mammalian populations in the Philippine archipelago. Journal of Biogeography, 32(2), 229–247. 10.1111/j.1365-2699.2004.01120.x

[eva13133-bib-0030] Hitchcock, H. B. , Keen, R. , & Kurta, A. (1984). Survival Rates of *Myotis leibii* and *Eptesicus fuscus* in Southeastern Ontario. Journal of Mammalogy, 65(1), 126–130. 10.2307/1381210

[eva13133-bib-1004] Howell, P. E. , Delgado, M. L. , & Scribner, K. T. (2017). Landscape genetic analysis of co‐distributed white‐footed mice (*Peromyscus leucopus*) and prairie deer mice (*Peromyscus maniculatus bairdii*) in an agroecosystem. Journal of Mammalogy, 98(3), 793–803.

[eva13133-bib-0031] Hua, P. , Zhang, L. , Guo, T. , Flanders, J. , & Zhang, S. (2013). Dispersal, mating events and fine‐scale genetic structure in the lesser flat‐headed bats. PLoS One, 8(1), e54428 10.1371/journal.pone.0054428 23349888 PMC3548791

[eva13133-bib-0032] Hufbauer, R. A. , Szűcs, M. , Kasyon, E. , Youngberg, C. , Koontz, M. J. , Richards, C. , Tuff, T. Y. , & Melbourne, B. A. (2015). Three types of rescue can avert extinction in a changing environment. Proceedings of the National Academy of Sciences of the United States of America, 112(33), 10557–10562. 10.1073/pnas.1504732112 26240320 PMC4547288

[eva13133-bib-0033] Hulme‐Beaman, A. , Dobney, K. , Cucchi, T. , & Searle, J. B. (2016). An ecological and evolutionary framework for commensalism in anthropogenic environments. Trends in Ecology and Evolution, 31, 633–645. 10.1016/j.tree.2016.05.001 27297117

[eva13133-bib-0034] Johnson, M. T. , & Munshi‐South, J. (2017). Evolution of life in urban environments. Science, 358, eaam8327 10.1126/science.aam8327 29097520

[eva13133-bib-0035] Jombart, T. , Devillard, S. , Dufour, A.‐B. , & Pontier, D. (2008). Revealing cryptic spatial patterns in genetic variability by a new multivariate method. Heredity, 101, 92–103. 10.1038/hdy.2008.34 18446182

[eva13133-bib-1005] Keller, L. F. , & Waller, D. M. (2002). Inbreeding effects in wild populations. Trends in Ecology and Evolution, 17, 230–241.

[eva13133-bib-0036] Kerth, G. , & Van Schaik, J. (2012). Causes and consequences of living in closed societies: Lessons from a long‐term socio‐genetic study on Bechstein's bats. Molecular Ecology, 21, 633–646. 10.1111/j.1365-294X.2011.05233.x 21883583

[eva13133-bib-0037] Kimura, M. , & Weiss, G. H. (1964). The stepping stone model of population structure and the decrease of genetic correlation with distance. Genetics, 49(4), 561–576. 17248204 10.1093/genetics/49.4.561 PMC1210594

[eva13133-bib-1007] Langmead, B. , & Salzberg, S. L. (2012). Fast gapped‐read alignment with Bowtie 2. Nature Methods, 9, 357–359. 22388286 10.1038/nmeth.1923 PMC3322381

[eva13133-bib-0038] Lenormand, T. (2002). Gene flow and the limits to natural selection. Trends in Ecology and Evolution, 17, 183–189. 10.1016/S0169-5347(02)02497-7

[eva13133-bib-0039] Li, H. , Parker, K. A. , & Kalcounis‐Rueppell, M. C. (2019). The luxury effect beyond cities: Bats respond to socioeconomic variation across landscapes. BMC Ecology, 19(1), 46 10.1186/s12898-019-0262-8 31676008 PMC6825354

[eva13133-bib-0040] Liu, X. , Huang, Y. , Xu, X. , Li, X. , Li, X. , Ciais, P. , Lin, P. , Gong, K. , Ziegler, A. D. , Chen, A. , Gong, P. , Chen, J. , Hu, G. , Chen, Y. , Wang, S. , Wu , Q. , Huang, K. , Estes, L. , & Zeng, Z. (2020). High‐spatiotemporal‐resolution mapping of global urban change from 1985 to 2015. Nature Sustainability, 3, 564–570.

[eva13133-bib-0041] Lowe, E. C. , Wilder, S. M. , & Hochuli, D. F. (2017). Life history of an urban‐tolerant spider shows resilience to anthropogenic habitat disturbance. Journal of Urban Ecology, 3(1), jux004 10.1093/jue/jux004

[eva13133-bib-0042] Luu, K. , Bazin, E. , & Blum, M. G. B. (2017) pcadapt: an R package to perform genome scans for selection based on principal component analysis. Molecular Ecology Resources, 17(1), 67–77. 27601374 10.1111/1755-0998.12592

[eva13133-bib-0043] McKinney, M. L. (2002). Urbanization, biodiversity, and conservation. BioScience, 52, 883–890. 10.1641/0006-3568(2002)052[0883:UBAC]2.0.CO;2

[eva13133-bib-0044] Medina, I. , Cooke, G. M. , & Ord, T. J. (2018). Walk, swim or fly? Locomotor mode predicts genetic differentiation in vertebrates (J‐M Gaillard, Ed,). Ecology Letters, 21, 638–645. 10.1111/ele.12930 29527800

[eva13133-bib-0045] Melero, Y. , Stefanescu, C. , Palmer, S. C. F. , Travis, J. M. J. , & Pino, J. (2020). The role of the urban landscape on species with contrasting dispersal ability: Insights from greening plans for Barcelona. Landscape and Urban Planning, 195, 103707 10.1016/j.landurbplan.2019.103707

[eva13133-bib-0046] Menke, S. B. , Guénard, B. , Sexton, J. O. , Weiser, M. D. , Dunn, R. R. , & Silverman, J. (2011). Urban areas may serve as habitat and corridors for dry‐adapted, heat tolerant species; an example from ants. Urban Ecosystems, 14(2), 135–163. 10.1007/s11252-010-0150-7

[eva13133-bib-0047] Miles, L. S. , Johnson, J. C. , Dyer, R. J. , & Verrelli, B. C. (2018). Urbanization as a facilitator of gene flow in a human health pest. Molecular Ecology, 27, 3219–3230. 10.1111/mec.14783 29972610

[eva13133-bib-0048] Miles, L. S. , Rivkin, L. R. , Johnson, M. T. J. , Munshi‐South, J. , & Verrelli, B. C. (2019). Gene flow and genetic drift in urban environments. Molecular Ecology, 28(18), 4138–4151. 10.1111/mec.15221 31482608

[eva13133-bib-1008] Mossman, C. A. , & Waser, P. M. (1999). Genetic detection of sex‐biased dispersal. Molecular Ecology, 8, 1063–1067. 10434424 10.1046/j.1365-294x.1999.00652.x

[eva13133-bib-0049] Munshi‐South, J. (2012). Urban landscape genetics: Canopy cover predicts gene flow between white‐footed mouse (*Peromyscus leucopus*) populations in New York City. Molecular Ecology, 21, 1360–1378. 22320856 10.1111/j.1365-294X.2012.05476.x

[eva13133-bib-0050] Munshi‐South, J. , & Kharchenko, K. (2010). Rapid, pervasive genetic differentiation of urban white‐footed mouse (*Peromyscus leucopus*) populations in New York City. Molecular Ecology, 19, 4242–4254. 10.1111/j.1365-294X.2010.04816.x 20819163

[eva13133-bib-1009] Munshi‐South, J. , & Richardson, J. L. (2020). Landscape genetic approaches to understanding movement and gene flow in Cities In Szulkin M., Munshi‐South J., & Charmantier A. (Eds.), Urban evolutionary biology. Oxford University Press.

[eva13133-bib-0051] Munshi‐South, J. , Zolnik, C. P. , & Harris, S. E. (2016). Population genomics of the Anthropocene: Urbanization is negatively associated with genome‐wide variation in white‐footed mouse populations. Evolutionary Applications, 9(4), 546–564. 10.1111/eva.12357 27099621 PMC4831458

[eva13133-bib-0052] Neubaum, D. J. , Wilson, K. R. , & O'shea, T. J. (2007). Urban maternity‐roost selection by big brown bats in Colorado. Journal of Wildlife Management, 71(3), 728–736. 10.2193/2005-684

[eva13133-bib-1010] Norberg, U. M. , & Rayner, J. M. V. (1987). Ecological morphology and flight in bats (Mammalia; Chiroptera): wing adaptations, flight performance, foraging strategy and echolocation. Philosophical Transactions of the Royal Society of London. B, Biological Sciences, 316, 335–427.

[eva13133-bib-0053] Norquay, K. J. O. , Martinez‐Nuñez, F. , Dubois, J. E. , Monson, K. M. , & Willis, C. K. R. (2013). Long‐distance movements of little brown bats (*Myotis lucifugus*). Journal of Mammalogy, 94(2), 506–515.

[eva13133-bib-1011] Olejniczak, M. J. , Spiering, D. J. , Potts, D. L. , & Warren, R. J. (2018). Urban forests form isolated archipelagos. Journal of Urban Ecology, 4.

[eva13133-bib-0054] Peres‐Neto, P. , & Galpern, P. (2015). Spatial pattern detection in genetic distance data using Moran's Eigenvector Maps. *R Package Version 1.0*.

[eva13133-bib-0055] Peterson, B. K. , Weber, J. N. , Kay, E. H. , Fisher, H. S. , & Hoekstra, H. E. (2012). Double digest RADseq: An inexpensive method for de novo SNP discovery and genotyping in model and non‐model species. PLoS One, 7, e37135 10.1371/journal.pone.0037135 22675423 PMC3365034

[eva13133-bib-1012] Petkova, D. , Novembre, J. , & Stephens, M. (2016). Visualizing spatial population structure with estimated effective migration surfaces. Nature Genetics, 48, 94–100. 26642242 10.1038/ng.3464 PMC4696895

[eva13133-bib-0056] Raj, A. , Stephens, M. , & Pritchard, J. K. (2014). FastSTRUCTURE: Variational inference of population structure in large SNP data sets. Genetics, 197(2), 573–589. 24700103 10.1534/genetics.114.164350 PMC4063916

[eva13133-bib-0057] Razgour, O. , Rebelo, H. , Puechmaille, S. J. , Juste, J. , Ibáñez, C. , Kiefer, A. , Burke, T. , Dawson, D. A. , & Jones, G. (2014). Scale‐dependent effects of landscape variables on gene flow and population structure in bats (C Burridge, Ed,). Diversity and Distributions, 20, 1173–1185. 10.1111/ddi.12200

[eva13133-bib-0058] Richardson, J. L. (2012). Divergent landscape effects on population connectivity in two co‐occurring amphibian species. Molecular Ecology, 21(18), 4437–4451. 10.1111/j.1365-294X.2012.05708.x 22891686

[eva13133-bib-0059] Richardson, J. L. , Brady, S. P. , Wang, I. J. , & Spear, S. F. (2016). Navigating the pitfalls and promise of landscape genetics. Molecular Ecology, 25(4), 849–863. 10.1111/mec.13527 26756865

[eva13133-bib-0060] Richardson, J. L. , Burak, M. K. , Hernandez, C. , Shirvell, J. M. , Mariani, C. , Carvalho‐Pereira, T. S. A. , Pertile, A. C. , Panti‐May, J. A. , Pedra, G. G. , Serrano, S. , Taylor, J. , Carvalho, M. , Rodrigues, G. , Costa, F. , Childs, J. E. , Ko, A. I. , & Caccone, A. (2017). Using fine scale spatial genetics of Norway rats to improve control efforts and reduce leptospirosis risk in urban slum environments. Evolutionary Applications, 10(4), 323–337. 10.1111/eva.12449 28352293 PMC5367079

[eva13133-bib-0061] Rochat, E. , Manel, S. , Deschamps‐Cottin, M. , Widmer, I. , & Joost, S. (2017). Persistence of butterfly populations in fragmented habitats along urban density gradients: Motility helps. Heredity, 119(5), 328–338. 10.1038/hdy.2017.40 28792492 PMC5637364

[eva13133-bib-0062] Rochette, N. C. , & Catchen, J. M. (2017). Deriving genotypes from RAD‐seq short‐read data using Stacks. Nature Protocols, 12(12), 2640–2659. 10.1038/nprot.2017.123 29189774

[eva13133-bib-0063] Rochette, N. C. , Rivera‐Colón, A. G. , & Catchen, J. M. (2019). Stacks 2: Analytical methods for paired‐end sequencing improve RADseq‐based population genomics. Molecular Ecology, 28(21), 4737–4754. 31550391 10.1111/mec.15253

[eva13133-bib-1013] Rohland, N. , & Reich, D. (2012). Cost‐effective, high‐throughput DNA sequencing libraries for multiplexed target capture. Genome Research, 22, 939–946. 22267522 10.1101/gr.128124.111 PMC3337438

[eva13133-bib-0064] Rossiter, S. J. , Zubaid, A. , Mohd‐adnan, A. , Struebig, M. J. , Kunz, T. H. , Gopal, S. , Petit, E. J. , & Kingston, T. (2012). Social organization and genetic structure: Insights from codistributed bat populations. Molecular Ecology, 21, 647–661. 10.1111/j.1365-294X.2011.05391.x 22168272

[eva13133-bib-0065] Santini, L. , González‐Suárez, M. , Russo, D. , Gonzalez‐Voyer, A. , von Hardenberg, A. , & Ancillotto, L. (2019). One strategy does not fit all: Determinants of urban adaptation in mammals. Ecology Letters, 22(2), 365–376. 10.1111/ele.13199 30575254 PMC7379640

[eva13133-bib-0066] Schmidt, C. , Domaratzki, M. , Kinnunen, R. P. , Bowman, J. , & Garroway, C. J. (2020). Continent‐wide effects of urbanization on bird and mammal genetic diversity. Proceedings of the Royal Society B‐Biological Sciences, 287, 20192497 10.1098/rspb.2019.2497 PMC7031673 32019443

[eva13133-bib-0067] Seto, K. C. , Güneralp, B. , & Hutyra, L. R. (2012). Global forecasts of urban expansion to 2030 and direct impacts on biodiversity and carbon pools. Proceedings of the National Academy of Sciences of the United States of America, 109, 16083–16088. 10.1073/pnas.1211658109 22988086 PMC3479537

[eva13133-bib-0068] Shirk, A. J. , & Cushman, S. A. (2011). sGD: Software for estimating spatially explicit indices of genetic diversity. Molecular Ecology Resources, 11, 922–934. 10.1111/j.1755-0998.2011.03035.x 21679313

[eva13133-bib-0069] Slatkin, M. (1987). Gene flow and the geographic structure of natural populations. Science, 236, 787–792. 10.1126/science.3576198 3576198

[eva13133-bib-0070] Stickel, L. F. (1968) Home range and travels In King J. A. (Ed.), Biology of Peromyscus (p. 411). American Society of Mammalogy.

[eva13133-bib-0071] Teferi, T. , & Millar, J. S. (1994). Effect of supplemental food on the dispersal of young *Peromyscus maniculatus* . Ecoscience, 1(2), 115–118.

[eva13133-bib-0072] Theodorou, P. , Radzevičiūtė, R. , Kahnt, B. , Soro, A. , Grosse, I. , & Paxton, R. J. (2018). Genome‐wide single nucleotide polymorphism scan suggests adaptation to urbanization in an important pollinator, the red‐tailed bumblebee (*Bombus lapidarius* L.). Proceedings of the Royal Society B: Biological Sciences, 285, 20172806. 10.1098/rspb.2017.2806 PMC5936727 29669900

[eva13133-bib-0073] Tsoar, A. , Shohami, D. , & Nathan, R. (2010). A movement ecology approach to study seed dispersal and plant invasion: An overview and application of seed dispersal by fruit bats In Richardson D. M. (Ed.), Fifty Years of Invasion Ecology (pp. 101–119). Wiley‐Blackwell.

[eva13133-bib-1014] Tucker, M. A. , Böhning‐Gaese, K. , Fagan, W. F. , Fryxell, J. M. , Van Moorter, B. , Alberts, S. C. , Ali, A. H. , Allen, A. M. , Attias, N. , Avgar, T. , Bartlam‐Brooks, H. , Bayarbaatar, B. , Belant, J. L. , Bertassoni, A. , Beyer, D. , Bidner, L. , van Beest, F. M. , Blake, S. , Blaum, N. , … Mueller, T. (2018). Moving in the Anthropocene: Global reductions in terrestrial mammalian movements. Science, 359, 466–469. 29371471 10.1126/science.aam9712

[eva13133-bib-0074] Turmelle, A. S. , Kunz, T. H. , & Sorenson, M. D. (2011). A tale of two genomes: Contrasting patterns of phylogeographic structure in a widely distributed bat. Molecular Ecology, 20(2), 357–375. 10.1111/j.1365-294X.2010.04947.x 21143331

[eva13133-bib-0075] Vangestel, C. , Mergeay, J. , Dawson, D. A. , Callens, T. , Vandomme, V. , & Lens, L. (2012). Genetic diversity and population structure in contemporary house sparrow populations along an urbanization gradient. Heredity, 109, 163–172. 10.1038/hdy.2012.26 22588131 PMC3424918

[eva13133-bib-0076] Walsh, B. , & Lynch, M. (2018). Interaction of selection, mutation, and drift In Walsh B., & Lynch M. (Eds.), Evolution and Selection of Quantitative Traits (pp. 175–205). Oxford University Press.

[eva13133-bib-0077] Wickham, H. (2009). ggplot2: Elegant Graphics for Data Analysis. Springer‐Verlag.

[eva13133-bib-0078] Withey, J. C. , & Marzluff, J. M. (2009). Multi‐scale use of lands providing anthropogenic resources by American Crows in an urbanizing landscape. Landscape Ecology, 24(2), 281–293. 10.1007/s10980-008-9305-9

[eva13133-bib-0079] Wood, B. C. , & Pullin, A. S. (2002). Persistence of species in a fragmented urban landscape: The importance of dispersal ability and habitat availability for grassland butterflies. Biodiversity and Conservation, 11, 1451–1468.

[eva13133-bib-0080] Wright, S. (1931). Evolution in Mendelian populations. Genetics, 16, 97–159. 17246615 10.1093/genetics/16.2.97 PMC1201091

[eva13133-bib-0081] Wright, S. (1943). Isolation by distance. Genetics, 28, 114–138. 17247074 10.1093/genetics/28.2.114 PMC1209196

[eva13133-bib-0082] Wright, S. (1951). The genetical structure of populations. Annals of Eugenics, 15, 323–354. 10.1111/j.1469-1809.1949.tb02451.x 24540312

[eva13133-bib-0083] Yauk, C. L. , & Quinn, J. S. (1996). Multilocus DNA fingerprinting reveals high rate of heritable genetic mutation in herring gulls nesting in an industrialized urban site. Proceedings of the National Academy of Sciences of the United States of America, 93(22), 12137–12141. 10.1073/pnas.93.22.12137 8901546 PMC37956

[eva13133-bib-0084] Yeaman, S. , & Otto, S. P. (2011). Establishment and maintenance of adaptive genetic divergence under migration, selection, and drift, 65(7), 2123–2129. Evolution. 10.1111/j.1558-5646.2011.01277.x 21729066

[eva13133-bib-1015] Zheng, X. , Levine, D. , Shen, J. , Gogarten, S. M. , Laurie, C. , & Weir, B. S. (2012). A high‐performance computing toolset for relatedness and principal component analysis of SNP data. Bioinformatics, 28, 3326–3328. 23060615 10.1093/bioinformatics/bts606 PMC3519454

