# Supplementary material for: Dispersal ability predicts spatial genetic structure in native mammals persisting across an urbanization gradient
Source: Evol Appl. 2020 Nov 6;14(1):163–77. doi: 10.1111/eva.13133 (PMC7819555; doi:10.1111/eva.13133)
Supplement: Supplementary file 1 — Supplementary Material [file EVA-14-163-s001.docx]

**Supporting Information**

**Table S1. Summary statistics pre- and post- filtering.**

| **Species** | **Pre-filtering** | | | | **Post filtering** | | |
| --- | --- | --- | --- | --- | --- | --- | --- |
|  | **N samples** | **Average number of raw reads per sample** | **Genotyped loci** | **Mean per sample coverage (stdev)** | **N samples** | **All SNPs** | **Single SNP per locus** |
| **Big brown bats** | **389** | **1,588,673** | **546099** | **46.6x (19.2x)** | **367** | **26736** | **4660** |
| **White-footed mice** | **215** | **xxx** | **3490848** | **xxx** | **215** | **206139** | **11788** |

**Figure S1**

Principal component analysis (PCA) showing that there was no clear genetic difference between museum mouse tissue samples and the samples collected by our lab. We therefore combined data from both sources for analyses.

**Figure S2**

fastStructure plots for mice (K=4) and bats (K=9).


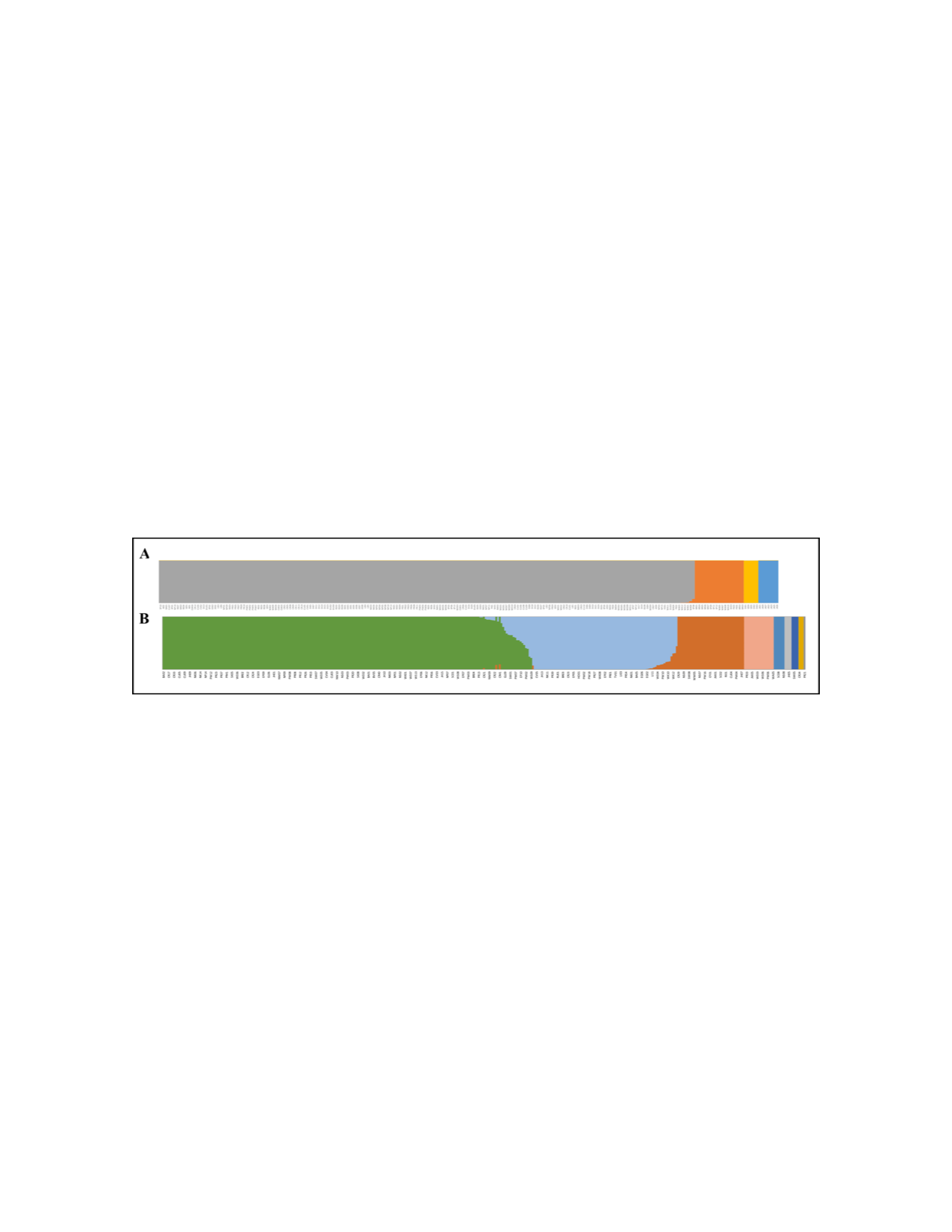


**Figure S3**

Observed heterozygosity of mice and bats obtained from sGD.

B

A


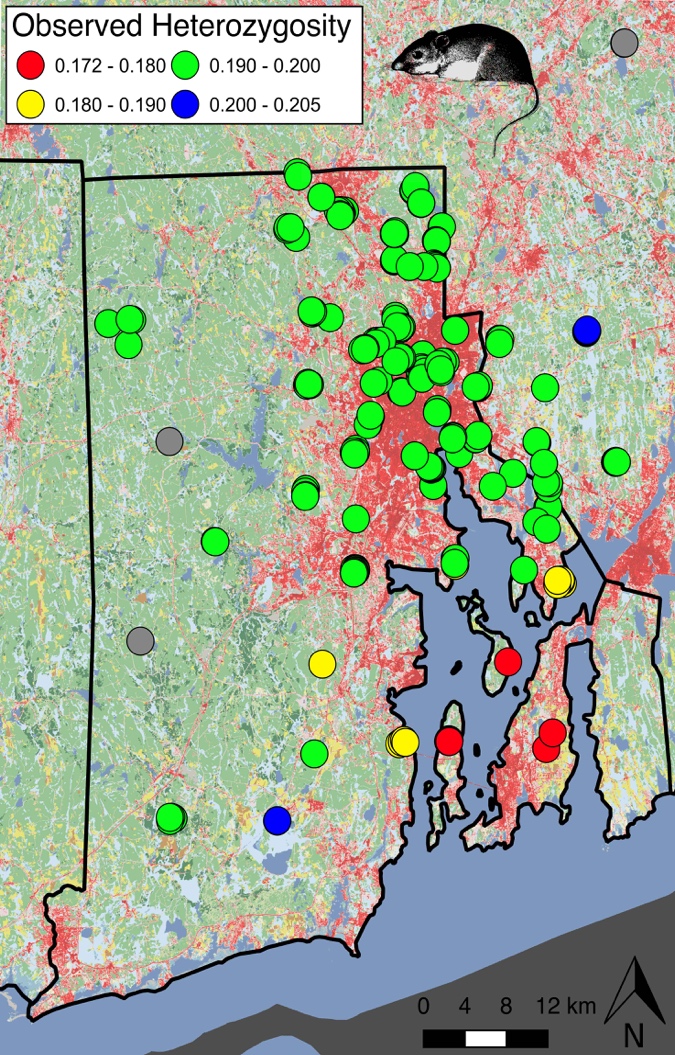

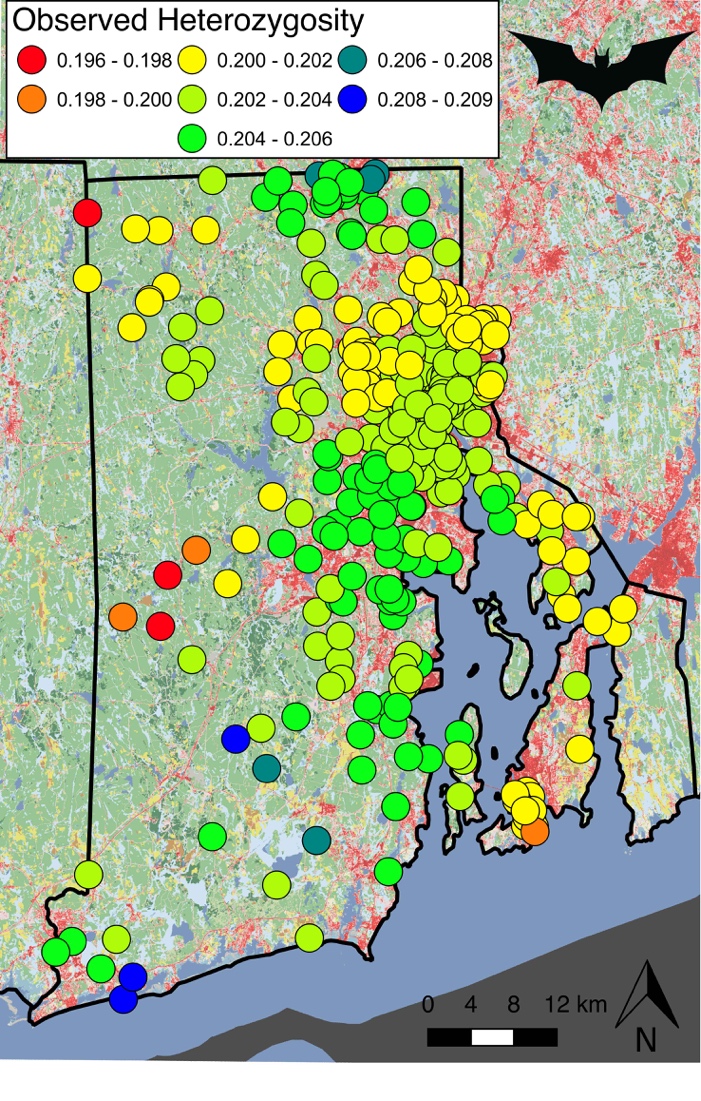


**Figure S4**: Principal component analyses plots for (A) mice and (B) bats. In each, the level of urbanization within a 500 meter radius around each sample is indicated by the color of the point (see legend in panel D).

**
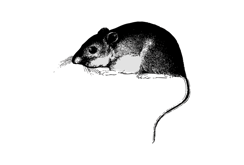

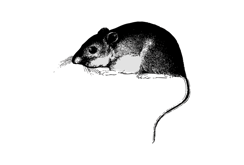
**
